# Supplementary material for: Preoperative Fasting and General Anaesthesia Alter the Plasma Proteome
Source: Cancers (Basel). 2020 Aug 27;12(9):2439. doi: 10.3390/cancers12092439 (PMC7564209; doi:10.3390/cancers12092439)
Supplement: Supplementary file 1 [file cancers-12-02439-s001.zip › cancers-908335 - SM conversion/cancers-908335 - Supplementary Figure.pdf]

cancers

Supplementary material

Preoperative Fasting and General Anaesthesia Alter the Plasma Proteome

Ulf Gyllensten, Sofia Bosdotter Enroth, Karin Stålberg, Karin Sundfeldt and Stefan Enroth

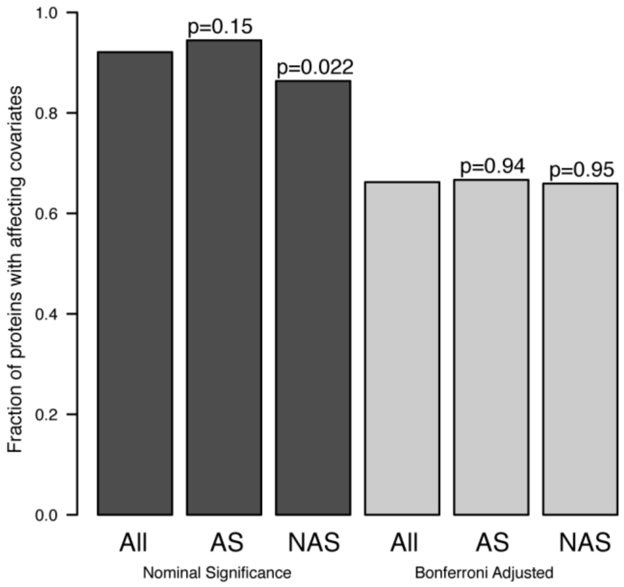

Figure S1: Fraction of proteins among all studied (labelled 'All') proteins, candidates for being affected by sedation ('AS') and those not affected by sedation ('NAS'). Dark grey bars represent nominally significance both in terms of effects of sedation and for covariates while light grey bars represent Bonferroni adjusted significance both for effects of sedation and covariates. P-values for statistical difference in fraction of affected proteins in the 'AS' or 'NAS' groups compared to 'All' were calculated using two-sided binominal tests with the fraction in 'All' as background fraction. Nominal p-values were rounded to 2 significant figures. List of effected covariates was taken from Enroth et al Scientific Reports, 2018, 8:5531.

© 2020 by the authors. Licensee MDPI, Basel, Switzerland. This article is an open access article distributed under the terms and conditions of the Creative Commons Attribution (CC BY) license (http://creativecommons.org/licenses/by/4.0/).
